# Higher Risks of Virologic Failure and All-Cause Deaths Among Older People Living with HIV in Chongqing, China

**DOI:** 10.1089/aid.2019.0096

**Published:** 2019-11-13

**Authors:** Guohui Wu, Chao Zhou, Xiangjun Zhang, Wei Zhang, Rongrong Lu, Lin Ouyang, Hui Xing, Yiming Shao, Yuhua Ruan, Han-Zhu Qian

**Affiliations:** ^1^ Chongqing Municipal Center for Disease Control and Prevention, Chongqing, China.; ^2^ School of Community Health Sciences, University of Nevada, Reno, Reno, Nevada.; ^3^ State Key Laboratory of Infectious Disease Prevention and Control (SKLID), Chinese Center for Disease Control and Prevention (China CDC), Collaborative Innovation Center for Diagnosis and Treatment of Infectious Diseases, Beijing, China.; ^4^ Shanghai Jiao Tong University-Yale Joint Center for Biostatistics and Data Science, School of Life Sciences and Biotechnology, Shanghai Jiao Tong University, Shanghai, China.; ^5^ Department of Biostatistics, Yale School of Public Health, New Haven, Connecticut.

**Keywords:** HIV, older people, antiretroviral therapy, virologic failure, all-cause mortality, China

## Abstract

Older people living with HIV (PLWH) may have delayed diagnosis and access to care and therefore have poorer disease outcomes. Little is known about HIV care and disease outcomes among older PLWH in China. This retrospective cohort study used data from all adult HIV/AIDS cases during 1988–2017 in Chongqing, China from two national databases. We compared demographic and behavioral profiles, HIV care, virologic suppression, and mortality between two age groups of 18–49 and ≥50 years. Multivariate logistic and cox regression analyses were used to calculate adjusted odds ratio (AOR) and adjusted hazard ratio (AHR) among older versus younger PLWH. Of 46,580 adult HIV/AIDS cases, 76.1% were men and 38.2% were 50 years of age or older. The proportion of older cases in men increased from 2.4% in 2002 to 51.8% in 2017, and in women from 3.3% to 57.9%. Older PLWH had a lower CD4 count than their younger counterparts at HIV diagnosis (median 323 vs. 391 cells/μL; *p* < .001). The average time from HIV diagnosis to initiation of antiretroviral therapy (ART) were 6.3 months among older and 12.8 months among younger PLWH (*p* < .001). Nearly one tenth (9.6%) had virologic failure within 12 months of ART initiation, and the odds of virologic failure among older PLWH was 80% higher [AOR 1.8; 95% confidence interval (CI), 1.1–3.0] than among younger ones after controlling for calendar year of initiating ART and other covariates. The mortality rate within 12 months of initiating ART was 9.8 deaths per 100 person years, and the risk of mortality among older PLWH was three times among younger ones (AHR, 3.1; 95% CI, 2.1–4.6). Older people represented an increasing proportion of new HIV/AIDS cases and were more likely to have virologic failure and mortality within 12 months of ART initiation.

## Introduction

HIV prevalence among people 50 years of age and older has been increasing steadily in the past two decades globally.^1,2^ The increase in the proportion of older individuals among people living with HIV (PLWH) is due primarily to the successes of highly active antiretroviral therapy (ART), which has prevented early deaths and extended the lives of individuals infected at a younger age.^3^ A smaller but increasing share of older adult cases are attributable to new infections in later life.^4–6^

Older PLWH may have different demographic and sociobehavioral profiles and clinical and immunological conditions, which affect outcomes of the disease.^7–11^ Both patients and clinicians often underestimate the risk of HIV among older adults, and this low-risk perception may cause delays in HIV testing and diagnosis.^12–14^ In addition, older PLWH face more challenges across stages of HIV care continuum due to later diagnosis and ART initiation in the disease course,^15,16^ a faster decline in immune function,^17^ and higher mortality rates,^6^ compared with younger people. For example, older age at ART initiation was associated with increased mortality in a large cohort of ART initiators in Latin America and the Caribbean.^9^

Current literature on HIV epidemic and care among older people is mainly from non-Asian countries. HIV epidemic in China is unique in some aspect. For example, it was initially driven by injecting drug use and contaminated plasma donation in the 1980s and 1990s, and since then it has transformed as heterosexual and homosexual transmissions. One notable feature of recent epidemic is high risk of homosexual transmission in young men, accounting for nearly 60% reported cases among the age group of 15–24 years in 2017. Another feature is rapid increase among men older than 60 years, with reported cases from 4,751 in 2010 to 19,815 in 2017 (unpublished data from China CDC). Yet few studies have investigated HIV issues among older Chinese people. A prospective cohort study among rural residents in southwestern China found that HIV incidence was 2.7 per 10,000 person years in all participants, but 71.3 among a subpopulation of males 50–69 years of age who had less than secondary schooling and were divorced or widowed.^5^ Another Chinese study found that older HIV-infected adults reported a lower level of wellbeing, higher level of depression, and poorer quality of life.^18^ A qualitative study found that older males had prevalent unsafe sex, and both male and female older adults had low awareness of HIV risk.^19^ To better understand the disease characteristics among older PLWH in China, we performed a retrospective cohort analysis of HIV epidemic, disease status, and access to care among all reported HIV/AIDS cases in Chongqing City, China.

## Methods

### Study site

Chongqing is the largest municipality that is directly administered by the central government of China and plays a key role in the development strategy for western China. It includes 25 districts and 13 counties, and has a total population of 30 million, including about 8.5 urban registered residents.

The first HIV infection was diagnosed in Chongqing in 1988, 3 years later than the first reporting of HIV/AIDS cases in 1985 in China. Between 2007 and 2012, the reported HIV infections grew at an average annual rate of 19.7%, substantially higher than the national rate (3.1%).^20^

### Data sources

Data were extracted from China's two web-based national databases for real-time collection and maintenance of information related to HIV case reporting and treatment, respectively. All newly identified HIV/AIDS cases from local hospitals, CDC clinics, and blood banks are reported through the HIV/AIDS case reporting system (CIS) at regular intervals, which includes demographic information and the date and venue of HIV diagnosis and the date and value of first CD4 test. The first CD4 test was typically performed immediately after HIV diagnosis. The HIV treatment system, called the HIV/AIDS Comprehensive Response Information Management System (CRIMS), has been described elsewhere,^21^ which includes information on ART regimens, viral load, comorbid diseases, loss to follow-up, transfer to other region, and death. Patients who initiated ART are followed at 15 days, 1 and 3 months, and then every 3 months, and their information on ART is updated in CIIMS accordingly. The recommended schedule for viral testing within 12 months of ART initiation is once every 1–2 months, but many patients do not follow the schedule. Designated staff at Chongqing Municipal Center for Disease Control and Prevention (Chongqing CDC), who have access to the records of HIV-infected individuals who are the residents of Chongqing City, downloaded the deidentified data from the online databases.

The study period covered from January 1, 1988 when the first case was reported in Chongqing City, to December 31, 2017. Because screening for inclusion was across the city and included all identified adult cases 18 years of age or older, sample size was not initially defined.

Variables used in this study included date of birth, date of HIV diagnosis, self-reported sexual orientation/most probable sources of HIV transmission, gender, death, CD4 count from CIS, HIV viral load, ART, and death and comorbidities from CRIMS. The inclusion criteria were: (1) registered resident in Chongqing; (2) 18 years of age or older; and (3) HIV-positive reported during January 1, 1988 and December 31, 2017. The data are anonymous and delinked from individual identifiable information.

### Ethics statement

This study utilizes anonymous data and data from secondary sources, which do not contain any personal identifiers. Therefore, ethics approval was not needed.

### Data analysis

The age of each participant was measured at the date of HIV diagnosis, which is also the time point of entering the cohort. Older people are defined as HIV-infected adults who were 50 years or older at HIV diagnosis.^22^ We calculated the proportions of older PLWH in all adult cases in each calendar year by sex.

We compared the demographic and behavioral characteristics between older (age ≥50) and younger PLWH (ages 18–49). We also compared HIV care between two age groups in men and women, respectively. Chi-square tests were used for categorical variables and t-tests were used for continuous variables.

We evaluated the difference of HIV treatment outcomes by age group, including virologic failure and mortality within 12 months of initiating ART. Considering the 6-month window of viral load testing, we assessed virologic failure up to 15 months after ART initiation for each participant. Multivariate logistic regression analysis was performed to calculate adjusted odds ratio and 95% confidence interval (CI) of virologic failure within 12 months of initiating ART among older age group while adjusting for potential confounders, including HIV transmission route and calendar year of initiating ART. Only those who had started ART and had viral load data within 12 months of ART initiation were included in this analysis. Viral load data were extracted from CRIMS until October 31, 2018. As older group had a higher proportion of heterosexual transmissions, the younger group had a higher proportion of homosexual transmissions, and an interaction term of age group and transmission route was included in the model. Cox regression analysis was performed to assess the relationship between older age and all-cause deaths within 12 months of initiating ART while adjusting for HIV transmission route and other covariates. Only those who had started ART and had information on survival status within 12 months of ART initiation were included in this analysis. As Chinese free ART programs started standardized treatment in 2008, one individual who started ART before 2008 was excluded from the analysis. Censorship was at the earliest recorded date of death, loss to follow-up, transfer out of the cohort, or up to 12 months of initiating ART. All analyses were performed in SPSS version 19.0.

## Results

### Trend of HIV epidemic among older adults in Chongqing

**FIG. 1.**
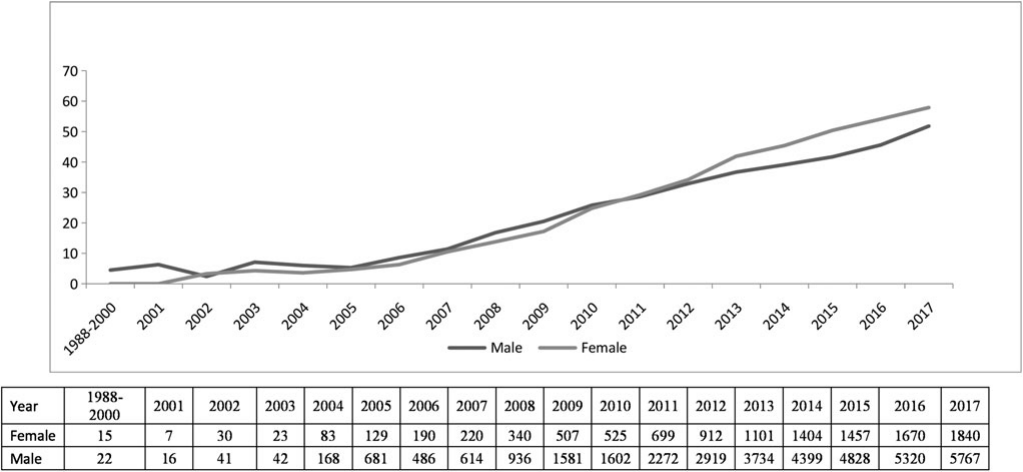
Proportion (%) of older (≥50 years) versus younger (18–49 years) HIV/AIDS cases by sex in Chongqing, China, 1988–2017. The table at the *bottom* of figure shows the number of new HIV/AIDS cases by sex and calendar year.

During years 1988–2017, a total of 46,580 adult HIV/AIDS cases were reported in Chongqing, and 76% were men and 24% were women (Table 1). Of all adult PLWH, 38.2% (*n* = 17,815) were 50 years of age or older. The average proportion of older cases in men was 37.2% (13,186/35,428) during 1988–2017, increasing from 2.4% in 2002 to 51.8% in 2017. The proportion of older cases in women was 41.5% (4,629/11,152) on average, increasing from 3.3% in 2002 to 57.9% in 2017 (Fig. 1).

**Table 1.** Comparison of Demographic and Behavioral Characteristics Among HIV-Infected Individuals Between Ages 18–49 and ≥50 Years in Chongqing, China, 1988–2017

	*Total,* N *(%)*	*Ages 18–49,* N *(%)*	*Age ≥50,* N *(%)*	p
Total	46,580 (100)	28,765 (61.8)	17,815 (38.2)	
Sex				<.001
Male	35,428 (76.1)	22,242 (77.3)	13,186 (74.0)	
Female	11,152 (23.9)	6,523 (22.7)	4,629 (26.0)	
Ethnicity				<.001
Han	45,258 (97.2)	27,749 (96.5)	17,509 (98.3)	
Other	1,322 (2.8)	1,016 (3.5)	306 (1.7)	
Marital status				<.001
Single	12,690 (27.2)	11,750 (40.9)	940 (5.3)	
Currently married	22,982 (49.3)	11,866 (41.3)	11,116 (62.4)	
Divorced or separated or widowed or other	10,113 (21.7)	4,532 (15.8)	5,581 (31.3)	
Unknown	795 (1.8)	617 (2.1)	178 (1.0)	
Education				<.001
Junior middle school or lower	32,352 (69.5)	16,576 (57.6)	15,776 (88.6)	
Senior high school	7,589 (16.3)	6,025 (21.0)	1,564 (8.8)	
College and above	6,378 (13.7)	5,924 (20.6)	454 (2.5)	
Unknown	261 (0.5)	240 (0.8)	21 (0.1)	
Employment				<.001
Employed	25,114 (53.9)	14,174 (49.3)	10,940 (61.4)	
Unemployed/retired	14,092 (30.3)	8,622 (30.0)	5,470 (30.7)	
Student	1,026 (2.2)	1,026 (3.6)	0 (0.0)	
Other	6,348 (13.6)	4,943 (17.2)	1,405 (7.9)	
Place of birth				<.001
Large city	17,652 (37.9)	12,043 (41.9)	5,609 (31.5)	
Medium city	18,945 (40.7)	10,526 (36.6)	8,419 (47.3)	
Small city	7,228 (15.5)	4,088 (14.2)	3,140 (17.6)	
Township/countryside	2,450 (5.3)	1,816 (6.3)	634 (3.6)	
Unknown	305 (0.7)	292 (1.0)	13 (0.1)	
Legal Chongqing Residency				<.001
Yes	43,866 (94.2)	26,432 (91.9)	17,434 (97.9)	
No	2,714 (5.8)	2,333 (8.1)	381 (2.1)	
Route of HIV infection				<.001
Injecting drug use	3,404 (7.3)	3,187 (11.1)	217 (1.2)	
Heterosexual contact	33,026 (70.9)	16,471 (57.3)	16,555 (92.9)	
Homosexual contact	9,055 (19.4)	8,285 (28.8)	770 (4.3)	
Other	1,095 (2.4)	822 (2.9)	273 (1.5)	
Calendar year of HIV diagnosis				<.001
1988–2003 (Pre ART)	195 (0.4)	188 (0.7)	7 (0.1)	
2004–2007 (No standardized ART)	2,571 (5.5)	23,69 (8.2)	202 (1.1)	
2008–2012 (Standardized ART)	12,293 (26.4)	9,008 (31.3)	3,285 (18.4)	
2013–2017 (TDF added)	31,521 (67.7)	17,200 (59.8)	14,321 (80.4)	

ART, antiretroviral therapy; TDF, tenofovir disoproxil fumarate.

### Demographic characteristics among older PLWH

The range of age was 18–49 and 50–91 in two age groups, and the mean (standard deviation) was 35.2 (8.8) and 62.0 (8.1), respectively. Table 1 compares the demographic and behavioral characteristics between two age groups. Most cases were ethnically Han for both older (98.3%) and younger groups (96.5%). Only 5% older but 40% younger PLWH were single, and older PLWH (31.3%) were more likely to be divorced or widowed than younger people (15.8%, *p* < .001). Older PLWH had a lower education level and a higher proportion of full-time employment than younger people. Compared with younger PLWH, older people were more likely to report heterosexual contact as HIV transmission route (92.9% vs. 57.3%, *p* < .001), and were less likely to report homosexual risk (4.3% vs. 28.8%, *p* < .001).

### HIV care among older PLWH

Chinese people can obtain free voluntary HIV testing at CDC clinics, and they may also get paid HIV testing in hospitals when they do medical exams or surgical operations. Less frequently, they can get a test through other ways, for example, buying testing kits online or at drug stores for self-testing. Of reported HIV/AIDS cases in Chongqing, the majority were diagnosed in hospital or CDC, and older cases were more likely to be diagnosed in hospital than younger cases (68.3% vs. 44%); this pattern of age group difference presents in both men and women. Older people had a lower CD4 count [median 323 cells/μL, interquartile range 196–429] than younger people (391, 264–501), and this pattern also presents in both men and women. About two third (67.8%) of adult PLWH were on ART, and there was no difference between older and younger men (69.9% vs. 65.8%, *p* > .05), but there was a slightly higher proportion among older women (72.3%) than younger women (67.8%, *p* < .05). The average time from HIV diagnosis to ART initiation were 6.3 months among older PLWH, which was about half for younger adults (12.8 months, *p* < .001); this difference was similar across all categories of CD4 count and in both men and women (Table 2).

**Table 2.** Comparison of HIV Care Between Age Groups of 18–49 and ≥50 Years in Chongqing, China, 1988–2017

	*All patients* (N = 46,580)			*Men* (N = 35,428)			*Women* (N = 11,152)		
	*Ages 18–49,* N *(%)*	*Age ≥50,* N *(%)*	P	*Ages 18–49,* N *(%)*	*Age ≥50,* N *(%)*	p	*Ages 18–49,* N *(%)*	*Age ≥50,* N *(%)*	p
Health care facility for making HIV diagnosis			<.001			<.01			<.001
Hospital	12,666 (44.0)	12,166 (68.3)		9,117 (41.0)	9,390 (71.2)		3,549 (54.4)	2,776 (57.5)	
CDC	14,292 (49.7)	5,025 (28.2)		11,610 (52.2)	3,310 (25.1)		2,682 (41.1)	1,715 (35.5)	
Other	1,807 (6.3)	624 (2.5)		1,515 (6.8)	486 (3.7)		292 (4.5)	13 8 (2.9)	
CD4 count at HIV diagnosis, cells/μL (median, IQR)			<.001			<.001			<.001
	391 (264–501)	323 (196–429)		391 (265–501)	314 (186–421)		389 (259–502)	348 (219–459)	
On ART			>0.05			>.05			<.05
Yes	19,519 (67.9)	12,042 (67.6)		15,095 (67.9)	8,499 (65.8)		4,424 (67.8)	3,543 (72.3)	
No	9,246 (32.1)	5,773 (32.4)		7,147 (32.1)	4,417 (34.2)		2,099 (32.2)	1,356 (27.8)	
Time from HIV diagnosis to ART initiation by CD4 count, months (mean ± SD)^a^									
Overall	12.8 ± 23.3	6.3 ± 14.5	<.001	12.5 ± 23.1	6.49 ± 14.8	<.001	13.7 ± 23.9	5.7 ± 13.7	<.001
0–199 (*n* = 12,039)	10.8 ± 21.9	4.3 ± 12.0		10.3 ± 21.3	4.4 ± 12.3		12.4 ± 23.4	4.2 ± 11.1	
200–349 (*n* = 10,653)	12.3 ± 21.5	6.1 ± 13.3		12.2 ± 21.4	6.6 ± 14.1		12.6 ± 21.6	5.0 ± 11.1	
≥350 (*n* = 5,788)	15.6 ± 25.8	8.8 ± 17.8		15.2 ± 25.6	9.3 ± 17.9		17.4 ± 26.6	7.9 ± 17.4	
Never tested (*n* = 3,081)	16.1 ± 27.9	10.6 ± 19.5		16.1 ± 28.1	10.8 ± 19.2		16.1 ± 27.6	9.9 ± 20.1	

^a^ Among 31,561 participants who have started ART.

IQR, interquartile range; SD, standard deviation.

### Comparison of virologic failure between older and younger PLWH

As Chinese free ART program did not standardize until 2008, we included participants who started ART since 2008 in this analysis. Of 25,693 participants who had started ART during 2008–2017 and were followed up within 12 months of initiating ART, 11,549 (45%) had information on viral load, including 46% (8,992/16,662) in younger and 43% (5,152/9,031) in older age groups. Of those having viral load data, nearly one tenth (9.6%) had virologic failure within 12 months of initiating ART, and this proportion was higher among older (11.9%) compared with younger people (8.5%, (*p* < .05). The odds of virologic failure for older PLWH was 80% higher (adjusted odd ratio, 1.8; 95% CI, 1.1–3.0) than younger ones after controlling for calendar year of initiating ART, HIV transmission route, and interaction term of age group by transmission route (Table 3).

**Table 3.** Comparison of HIV Virologic Failure Between Age Groups of 18–49 and ≥50 Years Within 12 (Range 9–15) Months of Initiating Antiretroviral Therapy in Chongqing, China, 2008–2017

	*No. of patients*	*No. of virologic failure (VL ≥200 copies/mL)*					
	N	n	% (95% CI)	*OR (95% CI)*	p	*AOR (95% CI)*^a^	p
Overall	11,548	1,114	9.6 (9.1–10.2)				
Age group, years							
18–49	7,670	653	8.5 (7.9–9.1)	Reference 1.0		Reference 1.0	
≥50	3,878	461	11.9 (10.9–12.9)	1.9 (1.2–3.1)	.006	1.8 (1.1–3.0)	<.05
Calendar year of initiating ART							
2008–2012 (Standardized ART)	1,255	274	21.8	Reference 1.0		Reference 1.0	
2013–2017 (TDF added)	10,293	840	8.2	0.3 (0.3–0.4)	<.001001	0.3 (0.3–0.4)	<.001
HIV transmission route							
Homosexual	3,065	172	5.6 (4.8–6.4)	Reference 1.0		Reference 1.0	
Heterosexual	7,642	878	11.5 (10.8–12.2)	2.2 (1.8–.6)	<.001	2.1 (1.7–2,6)	<.001
IDU	841	64	7.6 (5.8–9.4)	1.4 (0.9–2.0)	>.05	1.4 (1.0–2.1)	>.05
Age × transmission route							
Age × homosexual				Reference 1.0		Reference 1.0	
Age × heterosexual				1.7 (1.02–2.7)	<.05	1.4 (0.8–2.3)	>.05
Age × IDU				1.7 (0.8–3.3)	>.05	1.5 (0.8–3.1)	>.05

^a^ Adjusted for sex, marital status, CD4 count before ART initiation, WHO clinical stage before ART.

AOR, adjusted odds ratio; CI, confidence interval; IDU, injection drug use; OR, odds ratio; VL, viral load.

### Comparison of mortality between older and younger age groups

Among 29,241 adult PLWH who started ART and had data on survival status within 12 months of initiating ART, 2,732 deaths were observed in this time period, and the overall mortality rate was 9.8 deaths per 100 person years. Older PLWH had about twice mortality rate compared with younger ones (14.7% vs. 6.8%). The risk of mortality for older PLWH was three times among younger ones (adjusted hazard ratio, 3.1; 95% CI, 2.1–4.6) after controlling for calendar year of initiating ART, HIV transmission route, and interaction term of age group by transmission route (Table 4).

**Table 4.** Comparison of Mortality Rate Between Age Groups of 18–49 and ≥50 Years Within 12 Months of Initiating Antiretroviral Therapy in Chongqing, China, 2008–2017

	*No. of patients*	*No. of deaths*	*Person years*	*Deaths per 100 person years (95% CI)*	*HR (95% CI)*	p	*AHR (95% CI)*^a^	P
Overall	29,115	2,694	27,851	9.7 (9.3–10.0)				
Age group, years								
18–49	18,254	1,190	17,601	6.8 (6.4–7.1)	Reference 1.0		Reference 1.0	
≥50	10,861	1,504	10,250	14.7 (14.0–15.4)	3.8 (2.6–5.7)	<.001	3.1 (2.1–4.6)	<0.001
Calendar year of initiating ART								
2008–2012 (Standardized ART)	4,899	916	4,622.6	19.8 (18.7–21.0)	Reference 1.0		Reference 1.0	
2013–2017 (TDF added)	24,216	1,778	23,228.4	7.7 (7.3–8.0)	0.4 (0.4–0.4)	<.001	0.4 (0.4–0.5)	<0.001
HIV transmission route								
Homosexual	6,009	139	59,201	2.3 (2.0–2.7)	Reference 1.0		Reference 1.0	
Heterosexual	21,574	2,267	20,549	11.0 (10.6–11.5)	3.9 (3.2–4.8)	<.001	3.7 (3.0–4.5)	<0.001
IDU	1,532	288	1,382	20.8 (18.7–23.0)	11.1 (8.9–13.9)	<.001	7.7 (6.1–9.7)	<0.001
Age × transmission route								
Age × homosexual					Reference 1.0		Reference 1.0	
Age × heterosexual					1.9 (1.3–2.9)	.001	1.5 (1.0–2.3)	<0.054
Age × IDU					3.8 (2.1–6.8)	<.001	2.4 (1.3–4.4)	<0.01

^a^ Adjusted for sex, marital status, CD4 count before ART initiation, WHO clinical stage before ART.

AHR, adjusted hazard ratio; HR, hazard ratio.

## Discussion

Our study showed that older PLWH had higher level of virologic failure and mortality within the first year of initiating ART than younger people. Later diagnosis of HIV infection may contribute to poorer disease outcomes among older people, as shown by lower CD4 count at HIV diagnosis in this age group. These findings are consistent with study findings in other countries.^6,14–16^ Another reason may be the delay in ART initiation: older people started ART in about half year of HIV diagnosis, which is half as 12 months among younger people. Older people may also have worse responses to HIV treatment, and this should be evaluated in future research.

The proportion of older people among all reported adult cases in Chongqing City has been increasing over years; it was greater than 50% in both male and female cases in 2017. It is suggested that HIV prevention and treatment programs should set priorities for older PLWH. Lower education and risk awareness levels among older PLWH may post challenges for effective implementation of HIV prevention and care.^5^

Compared with younger adult cases, older PLWH in Chongqing were more likely to be divorced and had heterosexual transmission. These profiles of HIV epidemic in Chongqing reflect the epidemic across China. An increased number of HIV infections has been reported among youths nationwide in recent years, particularly among young men who have sex with men.

The study has limitations. First, disease progression, including virologic failure and mortality was assessed within 12 months of ART initiation. The longer progression is unknown and should be further studied. Second, we used all-cause mortality within 12 months of ART initiation as a variable of measuring age-specific effect of ART treatment. The older age group might have more non-AIDS-related deaths than the younger group, and this may lead to biased estimation of the age-specific effect of HIV treatment. However, this effect of non-AIDS-related death is unlikely to be significant, as we measured the mortality in a short time period. Third, due to limited available information in public health databases, other factors such as ART regimens and ART compliance, which may influence disease outcomes, were not assessed in this study. A prospective cohort study design for assessing potential confounding factors could overcome this limitation.

Despite these limitations, this is the first study of evaluating HIV treatment outcomes among older PLWH in China. It provides valuable information on demographic and behavioral profiles, access to HIV care, and disease progression among older PLWH. As this study included a large sample enrolled over a span of three decades and there are similarities in HIV epidemic profiles and HIV care between Chongqing and China, including the same HIV testing and ART guidelines implemented across the country, increasing proportion of older people, high proportion of heterosexual transmission among older PLWH, the study results may be generalizable to HIV-infected populations in and beyond China. As over half of newly reported HIV/AIDS in 2017 were older people in Chongqing, it poses urgency and challenges for the HIV prevention and treatment programs in Chongqing and other Chinese cities to provide high-quality service to older patients.
